# Binding Out of Relative Clauses in Native and Non-native Sentence Comprehension

**DOI:** 10.1007/s10936-022-09845-z

**Published:** 2022-02-22

**Authors:** Claudia Felser, Janna-Deborah Drummer

**Affiliations:** Potsdam Research Institute for Multilingualism, University of Potsdam, Karl-Liebknecht-Strasse 24-25, 14476 Potsdam, Germany

**Keywords:** Pronoun binding, c-command, Eye-movement monitoring, Non-native language processing, German

## Abstract

Pronouns can sometimes covary with a non c-commanding quantifier phrase (QP). To obtain such 'telescoping' readings, a semantic representation must be computed in which the QP's semantic scope extends beyond its surface scope. Non-native speakers have been claimed to have more difficulty than native speakers deriving such non-isomorphic syntax-semantics mappings, but evidence from processing studies is scarce. We report the results from an eye-movement monitoring experiment and an offline questionnaire investigating whether native and non-native speakers of German can link personal pronouns to non c-commanding QPs inside relative clauses. Our results show that both participant groups were able to obtain telescoping readings offline, but only the native speakers showed evidence of forming telescoping dependencies during incremental parsing. During processing the non-native speakers focused on a discourse-prominent, non-quantified alternative antecedent instead. The observed group differences indicate that non-native comprehenders have more difficulty than native comprehenders computing scope-shifted representations in real time.

## Introduction

The availability of a bound variable reading for pronouns normally requires the binder to c-command the pronoun (Reinhart, 1983), but exceptions to this requirement have frequently been noted (e.g. Barker, 2012).^1^ For a pronoun to covary with a non c-commanding quantifier phrase (QP)—a phenomenon known as 'telescoping' (Roberts, 1989)—is also sometimes possible for QPs located within tensed relative clauses. This is illustrated by the examples in (1)-(3) below.

**Table:** 

(1)	The one woman whom every true Englishman honours above all other women is his								
	mother. (Evans, 1977: 782)								
(2)	The grade that each student receives is recorded in his file. (Barker, 2012: 624)								
(3)	*Der*	*Dozent,*		*der*	*fast*	*jede*	*Studentin*		*faszinierte,*
	the	lecturer	_NOM_	that_NOM_	almost	every	student_NOM/ACC_		fascinated
	*las*	*ihren*	*Text*	*nochmal*	*Korrektur.*				
	read	her	text	again	proof				
	'The lecturer who fascinated almost every student proofread her text again.'								
					(German; Radó et al., 2019: 406)				

In examples (1) and (2), the pronoun *his* can be interpreted as covarying with the universally quantified noun phrases *every true Englishman* and *each student* respectively, even though both of these QPs are embedded within a relative clause (RC). Similarly, in example (3) from German, the possessive pronoun *ihren* ('her') can covary with the embedded object QP *jede Studentin* ('every student'). Evidence from offline tasks suggests that telescoping interpretations are readily available to native language (L1) comprehenders (e.g. Moulton & Han, 2018; Radó et al., 2019), and sentences containing a pronoun linked to a non c-commanding rather than to a c-commanding QP do not necessarily give rise to increased processing cost during L1 comprehension (Carminati et al., 2002). Several recent reading-time studies have found no evidence for telescoping dependencies being computed during real-time processing, however (Cunnings et al., 2015; Kush et al., 2015; Moulton & Han, 2018).

On the assumption that pronoun binding requires the binder to take scope over the pronoun, to obtain a telescoping reading a semantic representation must be computed in which the QP's semantic scope does not correspond to its surface scope. To account for the possibility of telescoping out of RCs, a syntactic approach to resolving this kind of syntax-semantics mismatch might assume that the embedded QP undergoes quantifier raising (QR) into the matrix clause (e.g. Barker, 2021; Hulsey & Sauerland, 2006). This operation is illustrated for example (2) in (4) below, where the QP *each student* has moved covertly out of the RC to adjoin to the matrix I(nflection)P(hrase) (May, 1985), as a result of which it now c-commands the pronoun *his*.^2^
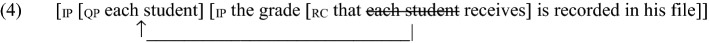


Alternative semantic approaches have been proposed that do not require QR, however (e.g. Sternefeld, 2019). Rather than seeking to adjudicate between different formal approaches to telescoping, the current study asks how readily telescoping readings are computed and at what point during comprehension non c-commanding quantified expressions are considered as antecedents for a pronoun.

Syntax-semantics mismatches such as those exemplified by (1–3) above also provide useful test cases for the hypothesis that second language (L2) speakers have problems re-accessing or manipulating previously built syntactic representations during anaphor resolution (Felser, 2016, 2019). L2 speakers have been reported to have difficulty deriving semantic or pragmatic representations that are non-isomorphic to a sentence's syntactic form (e.g. Boxell et al., 2017; Chu et al., 2014; Ionin et al., 2014; Wu & Ionin, 2019), albeit with the majority of studies informed by data from offline tasks. Assuming that the computation of telescoping readings in sentences such as (1–3) requires extending the QP's scope beyond the clause that contains it, we might expect L2 speakers to have more difficulty computing such readings compared to L1 speakers.

The rationale for the current study is twofold: (i) to re-examine previous claims to the effect that telescoping dependencies are not computed during real-time processing, and (ii) to test the hypothesis that L2 comprehenders have more difficulty than native speakers deriving non-isomorphic syntax-semantics mappings.

## Pronoun Binding and C-command in L1 Processing

Few studies to date have examined telescoping experimentally. Their results show that although telescoping readings are relatively easy to obtain in L1 comprehension (Carminati et al., 2002; Moulton & Han, 2018; Radó et al., 2019), pronouns do not seem to be linked to non c-commanding QPs during real-time incremental processing (Cunnings et al., 2015; Kush et al., 2015; Moulton & Han, 2018).

Using eye-movement monitoring during reading, Cunnings et al., (2015, experiment 2), for example, had native English speakers read sentences such as (4a, b), in which a pronoun was preceded by two potential antecedents, a c-commanding definite noun phrase (DP) (e.g. *the surgeon*) and a universally quantified noun phrase that either c-commanded the pronoun (5a) or not (5b). The gender-match between the pronoun and the QP was manipulated in order to assess whether participants would try to link the pronoun (here, *he*) to the QP.

**Table:** 

(5)	a	The surgeon saw that every old {man/woman} on the emergency ward silently wished that he could go a little bit faster.
	b	The surgeon who every old {man/woman} on the emergency ward saw silently wished that he could go a little bit faster.

The gender-mismatch paradigm (e.g. Sturt, 2003) is a frequently used diagnostic for referential dependency formation, as readers' attempts to link a pronominal expression to a potential antecedent are typically reflected in elevated reading times if the gender of the antecedent being retrieved is found to mismatch the pronoun's gender. Cunnings et al.'s (2015) analysis of the eye-movement data revealed effects of the gender-match between the QP and the pronoun for sentences such as (5a), indicating that participants tried to link the pronoun *he* to a c-commanding QP. No QP gender effects were observed for non c-commanding QPs (5b), however. The results from a follow-up experiment (experiment 3) showed that comprehenders had no difficulty linking pronouns to non c-commanding antecedents as such, if these were unquantified DPs. Another follow-up experiment (experiment 4) replicated the findings from experiment 2 for sentences of type (5b), although here a delayed effect of the QP's gender was observed during participants' reading of the final sentence region.

Cunnings et al.'s (2015) findings suggest that pronouns are not linked to non c-commanding QPs during processing. This conclusion is supported by the results from reading-time studies reported by Kush et al. (2015) and Moulton and Han (2018), who did not find any QP gender effects for negative QPs embedded within RCs (6a) (from Kush et al., 2015: 29) or QPs in sentence-initial adjunct clauses (6b) (from Moulton & Han, 2018: 199).

**Table:** 

(6)	a	The troop leaders that no {girl/boy} scout had respect for had scolded her after the incident at scout camp.
	b	After each boy brought fresh water from the kitchen quickly it seems that {he/she} went on an early break.

The absence of gender-mismatch effects for non c-commanding QPs in these studies may indicate that telescoping readings, to the extent that they are available, are computed at later stages of comprehension rather than during incremental processing. They may be derived in the course of sentence-final wrap-up processes (Just & Carpenter, 1980) or reflect the outcome of conscious reasoning. As c-commanding QPs reliably trigger gender effects, the lack of telescoping effects in the above studies is unlikely to reflect a general dispreference for linking pronouns to quantified antecedents.

Notice that in all previous studies that failed to find gender effects for non c-commanding QPs, the QP served as the subject of the clause containing it. Radó et al. (2019: 411) note that from the perspective of a QR-based syntactic approach to telescoping, raising an embedded subject is problematic as this should incur a violation of the empty category principle (ECP).^3^ Radó et al. used a self-paced reading task to investigate whether a subject/object asymmetry could be observed during the processing of German sentences such as (7a, b).

**Table:** 

(7)	a	*Seine*	*Ärztin,*		*die*		*jeden*		*Patienten*	
		his	doctor_NOM/ACC_		who_NOM/ACC_		every_ACC_		patient_ACC_	
		*seit*	*Langem*	*gekannt*		*hat,*	*hat*	*ihm*		*ein*
		since	long	known		has	has	him_DAT_		an
		*teures*	*Medikament*		*verschrieben*					
		expensive	medication		prescribed					
		'His doctor who has known every patient for a long time prescribed him an								
		expensive medication.'								
	b	*Seine*	*Ärztin,*		*die*		*jeder*		*Patient*	
		his	doctor_NOM/ACC_		who_NOM/ACC_		every_NOM_		patient_NOM_	
		*seit*	*Langem*	*gekannt*		*hat,*	*hat*	*ihm*		*ein*
		since	long	known		has	has	him_DAT_		an
		*teures*	*Medikament*		*verschrieben*					
		expensive	medication		prescribed					
		'His doctor who every patient has known for a long time prescribed him an								
		expensive medication.'								

In (7a) the accusative-marked QP *jeden Patienten* ('every patient') is the direct object of the embedded participle *gekannt* ('known'), whilst in (7b) the nominative-marked QP functions as its subject. In two additional experimental conditions the cataphoric possessive pronoun *seine* ('his') was replaced by the definite determiner *die* ('the'). If raising the QP into the matrix clause is possible in (7a) but not in (7b), then the cataphoric pronoun *seine* ('his') should have a bound reading in (7a) but not in (7b) as revealed by end-of-trial comprehension responses. Radó et al. (2019) additionally predicted longer RC reading times for (7b) compared to (7a) reflecting an attempted ECP violation in (7b).

There were no significant differences between the subject and object QP conditions in the proportions of times a bound reading for the pronouns *ihm* ('him') or *seine* (his') was permitted (around 30% on average). The authors did observe some reading-time differences between their experimental conditions, however. Sentences such as (7b) containing a subject QP caused measurable processing difficulty during the RC region, whereas at the pronoun *ihm* ('him') reading was slowed when the pronoun was preceded by an object QP (7a) compared to a subject QP (7b). Whilst Radó et al.'s (2019) reading-time results are difficult to interpret, the authors interpret the absence of any subject/object asymmetries in the availability of telescoping readings as evidence against syntactic approaches to telescoping.

In sum, previous studies on telescoping out of RCs have shown that even though native comprehenders can obtain bound readings for non c-commanded pronouns relatively easily, they do not seem to link pronouns to non c-commanding subject QPs during processing. Considering the possibility that extending the scope of a subject QP might be more difficult than extending the scope of an object QP, the materials used in our reading-time experiment include stimulus sentences with embedded object QPs.

## Quantification and Binding in L2 Processing

To the best of our knowledge, the availability of telescoping readings in L2 comprehension has never been investigated experimentally. Some previous findings reported in the L2 processing literature are potentially relevant to the current study, however.

First, there is evidence suggesting that L2 comprehenders disprefer linking pronouns to quantified antecedents even if these c-command the pronoun, showing a preference for resolving pronouns via coreference assignment instead. Unlike binding, coreference dependencies do not require c-command and are thought to be mediated by discourse-level representations (e.g. Grodzinsky & Reinhart, 1993). Trompelt and Felser (2014) carried out an offline questionnaire and an eye-movement monitoring experiment to investigate whether, in sentences such as (8) below, L1 and L2 German speakers would link the pronoun *er* ('he') to a c-commanding QP (*jeder Maurer* 'every bricklayer') or to a non c-commanding coreference antecedent (*Georg*).

**Table:** 

(8)	*Jeder*	*Maurer,*	*der*	*sah,*	*dass*	*Georg*	*auf*	*der*	*Baustelle*	*war,*
	every	bricklayer	who	saw	that	G.	on	the	building.site	was
	*ahnte,*	*dass*	*er*	*heute*	*fleißig*	*arbeiten*		*muss.*		
	suspected	that	he	today	hard	work		must		
	‘Every bricklayer who saw that George was on the building site suspected that he had									
	to work hard today.’									

The questionnaire results showed that, while both participant groups ultimately preferred the coreference antecedent over the QP antecedent, this preference was significantly stronger in the L2 group. The analysis of the eye-movement data revealed no clear antecedent preference in the L1 group, but a preference for linking the pronoun to the named antecedent during processing in the L2 group. A dispreference for linking singular pronouns to universally quantified antecedents may be due to the fact that even though such QPs are grammatically singular, they are conceptually plural, or because QPs are non-referential. It may be either these factors or a more general preference for coreference over binding that prevented L2 speakers from linking the pronoun to the QP antecedent in Trompelt and Felser's (2014) study.

Secondly, L2 comprehenders appear to resolve syntax-semantics mismatches in a non-nativelike way during processing. Boxell et al. (2017), for example, used eye-movement monitoring during reading to investigate the processing of antecedent-contained deletion (ACD) structures as in (9) by L1 and L2 speakers of English. ACD involves a special case of verb-phrase (VP) ellipsis in which the elided VP is contained within its own antecedent verb phrase, rendering the VP gap recursive.

**Table:** 

(9)	The scout was taught to [_VP_ catch {*every/the*} *snake that the explorer did* [_VP_ __]] in the outback in the strong heat

For ACD sentences to be interpretable, a semantic or discourse representation must be derived from a sentence's surface form such that recovering the elided material does not result in a recursive loop (e.g. via QR). In Boxell et al.'s (2017) study only the L1 speakers showed evidence of ACD processing being facilitated by quantified vs. non-quantified noun phrases, as would be expected if QR were involved in ACD resolution, but not the L2 group.

Difficulty extending the surface scope of quantificational expressions should also affect comprehenders' interpretation of scope ambiguities. L2 speakers have indeed been reported to have difficulty computing inverse scope readings for sentences such as *Every horse did not jump over the fence* (e.g. Wu & Ionin, 2019). Reading-time results show that the processing of scope ambiguities can remain non-nativelike even at higher levels of L2 proficiency (e.g. Lee, 2009).

Thirdly, L2 comprehenders have been found to retrieve syntactically or discourse-prominent antecedents for pronominal elements during processing when L1 speakers do not (e.g. Patterson et al., 2014), and even if these antecedents are grammatically inappropriate. Felser and Cunnings (2012), for example, observed L2 speakers linking reflexives to grammatically inappropriate but discourse-prominent antecedents during online reading, and Kim et al. (2015) found L2 speakers linking non-reflexive object pronouns to grammatically inappropriate local subjects (i.e. *Mickey*) when hearing spoken instructions such as *Look at Goofy. Have Mickey touch him.*

The above seemingly diverse set of findings may in fact be attributable to the same underlying cause: greater difficulty re-accessing and/or manipulating previously built structural representations during L2 than during L1 comprehension, combined with a relatively stronger reliance on discourse-level cues to interpretation. This could be because L2 structural representations lack sufficient detail to start with (compare Clahsen & Felser's, 2006, 2018 shallow structure hypothesis), or because they are unstable and fade too quickly from memory to be able to serve as the input to further derivational (e.g. scope shifting) processes (Felser, 2019). Syntactically mediated binding will then be eschewed in favour of discourse-based coreference assignment because establishing binding relationships requires navigating previously built syntactic representations and retracing c-command paths. L2 speakers' relatively stronger attraction towards discourse-prominent or coreference antecedents (e.g. Trompelt & Felser, 2014) may simply reflect an alternative, discourse-based antecedent search strategy.

Note that some of the above findings, specifically L2 speakers' consideration of grammatically inappropriate antecedents, can also be accounted for by Cunnings (2017) memory interference hypothesis, according to which L2 speakers show increased susceptibility to memory interference during processing. The current study was not designed to put this hypothesis to the test, however. Here we examine the hypothesis that L2 speakers have more difficulty than L1 speakers deriving semantic representations that cannot be read off a sentence's surface syntactic form.

## Experiment 1

Our first experiment was an offline antecedent evaluation task investigating whether L1 and L2 speakers of German allow for pronouns to be linked to non c-commanding QPs inside relative clauses. The questionnaire experiment consisted of two parts. Experiment 1A examined the availability of telescoping readings for pronouns preceded by embedded subject or object QPs, and Experiment 1B was a control experiment to establish how readily pronouns were linked to c-commanding QPs and to non c-commanding coreference antecedents. A full list of our experimental materials for Experiments 1 & 2 and the full statistical model outputs and formulae for both experiments are available at the Center for Open Science Framework website at https://osf.io/b3duv/.

### Method

#### Participants

Forty native speakers of German (5 male) with a mean age of 26.8 years (SD: 8.2, range: 18–61) and 43 native Russian speakers took part in Experiment 1.^4^ They were recruited via the institute’s participant database, the cognitive sciences participant pool SONA at the University of Potsdam, and social media. The data from two Russian speakers were later excluded due to their low response accuracy on the filler items. The remaining 41 Russian speakers (6 male) had a mean age of 31.0 (SD: 9, range: 20–58) and had started learning German as a second or foreign language at the age of 15.4 on average (SD: 6.8, range: 6–35), mostly in a formal educational setting. The L2 participants self-rated their German language skills (reading, listening, writing and speaking) on a seven-point scale from 'very poor' to ‘native-like’. The majority of ratings were near the top end of the scale, with average ratings across the four skills mostly falling within the 'good' (25.75%), 'very good' (47%) or 'native-like' (18.25%) categories. None of the L2 participants provided any ratings within the bottom-level categories of 'poor' or 'very poor'.

#### Materials

We created 12 sentence pairs each for Experiment 1A and Experiment 1B, with each experimental sentence containing a pronoun and two potential antecedents. An example stimulus pair for Experiment 1A is shown in (10a, b) below.

**Table:** 

(10)	a.	EMBEDDED OBJECT QP							
		*Der *	*Förster,*	*der*		*jeden*		*Gärtner*	*kannte,*
		*the*	*forester*	who_NOM_		every_ACC_		gardener	knew
		*war*	*überzeugt,*	*dass*	*er*	*mehr*	*Bäume*	*pflanzen*	
		was	convinced	that	he	more	trees	plant	
		*sollte.*							
		*should*							
		'The forester who knew every gardener was convinced that he should plant more trees.'							
	b.	EMBEDDED SUBJECT QP							
		*Der *	*Förster,*	*den*		*jeder*		*Gärtner*	*kannte,*
		*the*	*forester*	who_ACC_		every_NOM_		gardener	knew
		*war*	*überzeugt,*	*dass*	*er*	*mehr*	*Bäume*	*pflanzen*	
		was	convinced	that	he	more	trees	plant	
		*sollte.*							
		*should*							
		'The forester who every gardener knew was convinced that he should plant more trees.'							

Each sentence contained a definite noun phrase (DP) in matrix subject position (e.g. *der Förster* 'the forester') that was modified by an RC containing a universally quantified noun phrase (e.g. *jeder Gärtner* 'every gardener'). The QP either functioned as an object (10a) or as a subject (10b) but never c-commanded the pronoun. The masculine singular personal pronoun *er* 'he' was located in the subject position of a complement clause selected by the matrix predicate. The masculine form was chosen because unlike feminine or neuter singular pronouns, *er* is unambiguously marked for nominative case. Both the DP and the QP referred to people (by means of role nouns) and their grammatical features matched those of the pronoun.

The stimuli for Experiment 1B were created by reversing the syntactic positions of the DP and QP antecedents as shown in (11a, b).

**Table:** 

(11)	a.	EMBEDDED OBJECT DP							
		*Jeder*	*Gärtner,*	*der*		*den*		*Förster*	*kannte,*
		every	gardener	who_NOM_		the_ACC_		forester	knew
		*war*	*überzeugt,*	*dass*	*er*	*mehr*	*Bäume*	*pflanzen*	
		was	convinced	that	he	more	trees	plant	
		*sollte.*							
		should							
		‘Every gardener who knew the forester was convinced that he should plant more trees.'							
	b.	EMBEDDED SUBJECT DP							
		*Jeder*	*Gärtner,*	*den*		*der*		*Förster*	*kannte,*
		every	gardener	who_ACC_		the_NOM_		forester	knew
		*war*	*überzeugt,*	*dass*	*er*	*mehr*	*Bäume*	*pflanzen*	
		was	convinced	that	he	more	trees	plant	
		*sollte.*							
		should							
		‘Every gardener who the forester knew was convinced that he should plant more trees.'							

Here the QP in matrix subject position c-commands the pronoun and is thus able to bind it, whereas the DP inside the relative clause can serve as a coreference antecedent. The purpose of Experiment 1B was to examine whether participants might have a preference for coreference over binding, and whether embedded object antecedents are preferred over subject antecedents (or vice versa) independently of their quantificational status.

The experimental items from Experiment 1 were distributed across four lists in a Latin-square design and mixed with 36 filler sentences. Twelve of these sentences were pseudo-fillers, resembling the experimental sentences in the RC structure and containing different QP types in varying syntactic positions. Six unambiguous fillers were included to allow us to verify whether participants were performing the task attentively.

#### Procedure

The experiment was implemented as a web-based questionnaire conducted via the survey platform *SoSci survey* (Leiner, 2014). Each item was presented on an individual screen in black font (Arial sans-serif, 10pt) against a white background. Stimulus sentences were presented in bold with the critical pronoun underlined. Underneath each sentence a question was shown asking about potential antecedents for the pronoun ("He can refer to…"), pointing out that multiple options might be possible. The two sentence-internal candidate noun phrases were listed along with checkboxes. For each candidate antecedent participants had to indicate whether the pronoun could possibly refer to it by ticking the corresponding checkbox (*yes* response) or leaving it blank (*no* response). Three example items explained the task. Completing the questionnaire took participants around 15 min on average. Compensation was provided in the form of course credit.

Mixed-effects logistic regression analyses were conducted for each sub-experiment (1A & 1B) separately with the statistical software R version 3.6.0 (R Core Team, 2019) using the lmerTest package version 3.1.2 (Kuznetsova et al., 2017). The models contained Response (*yes* vs. *no*) as the dependent variable and in the fixed part the sum-coded factors Condition (embedded object vs. embedded subject) and Group (L1 vs. L2), their interaction and the centred factor Trial as a covariate. As Condition was sum-coded, neither level served as the reference level. The models' most complex random structures contained by-subject and by-item random slopes for Condition (Formula: Response ~ Condition * Group + Trial + (1 + Condition | Subj) + (1 + Condition | Item). Models with different random structures were computed and likelihood ratio tests helped to determine the model with the best fit at an alpha level of 0.05 (Baayen et al., 2008). Typically, the model with the simplest random structure (+ (1 | Subj) + (1 | Item)) was selected as more complex models did not converge or did not represent a better fit.

### Results

Table 1 provides an overview of participants' responses in Experiment 1A and 1B, and the between-group statistical analyses are summarized in Table 2. Positive estimates for Condition are associated with a higher number of *yes* responses in the embedded subject conditions (10/11b) as compared to the embedded object conditions (10/11a). Positive estimates for Group are associated with a higher number of *yes* responses in the L2 group as compared to the L1 group.

**Table 1:** Proportions (SEs) of 'yes' responses in Experiments 1A and 1B per group and condition

Experiment	Group	Condition	DP(1A)/QP(1B)	QP(1A)/DP(1B)
1A	L1	Embedded object QP	.88 (.03)	.43 (.05)
		Embedded subject QP	.93 (.02)	.23 (.04)
	L2	Embedded object QP	.81 (.04)	.37 (.04)
		Embedded subject QP	.87 (.03)	.31 (.04)
1B	L1	Embedded object DP	.55 (.05)	.82 (.04)
		Embedded subject DP	.73 (.04)	.70 (.04)
	L2	Embedded object DP	.67 (.04)	.70 (.04)
		Embedded subject DP	.72 (.04)	.64 (.04)

**Table 2:** Summary of the statistical between-group analyses for two potential antecedent noun phrases, Experiments 1A and 1B

		NP1: DP(1A)/QP(1B)				NP2: QP(1A)/DP(1B)			
Exp		Est	SE	*z*	*p*	Est	SE	*z*	*p*
1A	Intercept	**3.516**	**0.639**	**5.499**	** < 0.001**	**−0.820**	**0.140**	**−5.852**	** < 0.001**
	Condition	**0.438**	**0.190**	**2.229**	**0.022**	**−0.329**	**0.105**	**−3.128**	**0.002**
	Group	−0.428	0.305	−1.403	0.161	0.046	0.137	0.338	0.736
	Cond*Grp	0.008	0.185	0.043	0.966	0.193	0.105	1.843	0.065
1B	Intercept	**1.011**	**0.346**	**2.920**	**0.004**	**1.204**	**0.279**	**4.316**	** < 0.001**
	Condition	**0.375**	**0.115**	**3.264**	**0.001**	**−0.276**	**0.114**	**−2.416**	**0.016**
	Group	0.155	0.158	0.982	0.326	−0.282	0.155	−1.822	0.068
	Cond*Grp	−0.179	0.113	−1.582	0.114	0.108	0.113	0.955	0.339

*p* < .05 in bold

In Experiment 1A, DP antecedents elicited a high proportion of *yes* responses across both groups, but QP antecedents were also deemed acceptable around a third of the time on average. DP antecedents were accepted more frequently if the embedded QP was a subject, and QP antecedents were considered possible more frequently if they functioned as objects. To see whether our L1 and L2 participants' response patterns differed statistically, we first compared the two groups' responses across the two conditions for each of the two antecedents. For DP antecedents, we found a significant main effect of Condition that was not modulated by the factor Group, reflecting the fact that DP antecedents were accepted more readily if the embedded QP was in subject position compared to when it was in object position. For QP antecedents we found a main effect of Condition indicating that the embedded QP was selected significantly less often when it was a subject compared to when it was an object. A marginally significant Condition by Group interaction reflected the fact that the Condition effect was carried by the L1 group. Pairwise comparisons confirmed this by revealing a significant effect of Condition in the L1 group (Est. = −0.629, *z* = −3.570, *p* < 0.001) but not in the L2 group (Est. = −0.153, *z* = −0.969, *p* = 0.332).

In Experiment 1B both antecedents were considered possible about equally often on average. QPs in matrix subject position were deemed possible antecedents more often if the embedded DP was a subject, and DP antecedents were more likely to be considered possible antecedents if they functioned as objects. Our between-groups analyses yielded significant main effects of Condition in different directions for c-commanding QP and embedded DP antecedents. Although there were no significant interactions with the factor Group, the observed Condition effects were again carried by the L1 group.

### Discussion

Our first experiment's primary aim was to ascertain whether both L1 and L2 speakers are able to obtain telescoping readings for QPs inside relative clauses. The results from Experiment 1A confirm that both participant groups allowed for pronouns to covary with non c-commanding QPs around one-third of the time on average. This is in line with previous findings from L1 German speakers (Radó et al., 2019). The fact that our native Russian-speaking participants were also able to compute telescoping interpretations in German indicates that extending the QP's scope was not a problem for them as such.

The results from Experiment 1B show that the relatively lower proportion of *yes* responses to QP compared to DP antecedents in Experiment 1A was not due to participants being generally less likely to link pronouns to QP than to DP antecedents. They moreover confirm that there was no general dispreference for linking pronouns to non c-commanding antecedents. We also observed a subject/object asymmetry such that embedded antecedents were more likely to be deemed possible antecedents if they were objects rather than subjects, albeit significantly so only for L1 speakers. This indicates that both telescoping and coreference readings are more difficult to obtain for non c-commanding subject compared to object antecedents.

## Experiment 2

Having shown that both L1 and L2 comprehenders allow for pronouns to be linked to QPs inside relative clauses in Experiment 1A, we carried out an eye-movement-monitoring-during-reading experiment to examine whether (and if so, when) non c-commanding QP antecedents are considered during processing.

### Method

#### Participants

Participants were recruited from the University of Potsdam community and from the Berlin area. We tested 63 native German speakers, the data from three of whom were later excluded because of track loss. The remaining 60 participants' (22 male) mean age was 24.6 years (range: 19–38 years). The non-native participant group was comprised of 50 L2 speakers of German with Russian as their L1. The data from three L2 speakers were excluded because of track loss. The remaining 47 participants (7 male) had a mean age of 25.7 years (range: 18–38 years). They had started learning German between the ages of 6–25 years (mean: 13.4 years), and all of them had been living in Germany for at least six months at the time of testing (mean: 7.3 years, range: 0.5–23 years). To obtain an indication of our L2 participants' proficiency in German, they were asked to complete the web-based Goethe Institute Placement Test (Goethe Institute, 2010). Participants' mean Goethe test score was 25/30 points, with a range from 18 to 30 points. These scores placed them within the B2–C2 range according to the Common European Framework of Reference for Languages. All participants had normal or corrected-to-normal vision and provided their informed consent to participate in our study.

#### Materials

Our eye-movement experiment had a 2 × 2 design that was modelled after Cunnings et al.'s (2015) experiment 4, but with a QP located in object rather than subject position. Twenty-four stimulus quadruplets were constructed by manipulating the gender match between the personal pronoun *er* ('he') and two potential antecedents, a c-commanding DP and a non c-commanding QP. The sentence containing the critical pronoun was preceded by a short lead-in sentence which served to set the scene, and followed by a closing sentence. The four experimental conditions are exemplified by (12a–d) below.

**Table:** 

(12)	LEAD-IN SENTENCE:				*Der Schlosspark war riesig gross*.					
					'The castle park was extremely large.'					
	a.	DP Match, QP Match								
		*Der *	*König, *		*der *		*jeden *	*Gärtner *		*kannte, *
		the	king		who_NOM_		every	gardener_ACC-MASC_		knew
		*war*	*überzeugt, *	*dass *	*er *	*mehr *	*Bäume*		*pflanzen *	
		was	convinced	that	he	more	trees		plant	
		*sollte.*								
		should								
	b.	DP Match, QP Mismatch								
		*Der *	*König, *		*der *		*jede *	*Gärtnerin *		*kannte, *
		the	king		who_NOM_		every	gardener_NOM/ACC-FEM_		knew
		*war*	*überzeugt, *	*dass *	*er *	*mehr *	*Bäume*		*pflanzen *	
		was	convinced	that	he	more	trees		plant	
		*sollte.*								
		should								
	c.	DP Mismatch, QP Match								
		*Die*	*Königin, *		*die *		*jeden *	*Gärtner *		*kannte, *
		the	queen		who_NOM_		every	gardener_ACC-MASC_		knew
		*war*	*überzeugt, *	*dass *	*er *	*mehr *	*Bäume*		*pflanzen *	
		was	convinced	that	he	more	trees		plant	
		*sollte.*								
		should								
	d.	DP Mismatch, QP Mismatch								
		*Die *	*Königin, *		*die *		*jede *	*Gärtnerin *		*kannte, *
		the	queen		who_NOM_		every	gardener_NOM/ACC-FEM_		knew
		*war*	*überzeugt, *	*dass *	*er *	*mehr *	*Bäume*		*pflanzen *	
		was	convinced	that	he	more	trees		plant	
		*sollte.*								
		should								
	'The king/queen, who knew every (male/female) gardener, was convinced that he should plant more trees.'									
	CLOSING SENTENCE:					*Dann würde es mehr Vögel im Park geben.*				
						‘There would be more birds in the park then.’				

Our experimental sentences were structurally identical to the stimulus items from Experiment 1A containing object QPs (10a). The experimental items were distributed across different presentation lists in a Latin-square design and mixed with 140 fillers, 80 of which were short stimulus texts from two unrelated experiments, yielding 164 items per list in total. Sixteen of the critical and 33 of the filler items were followed by a *yes/no* comprehension question. The experimental sentences were spread across three lines such that the critical pronoun appeared roughly in the middle of the second line.

### Procedure

All participants were tested individually in a dedicated laboratory room. Their eye movements were recorded using an SR Research Eyelink 1000 system with a sampling rate of 1000 Hz. Although participants read binocularly, only their right eye was tracked unless calibration of the right eye was not possible. The stimulus texts were presented in black Courier New font (18pt) on a white background, and their presentation order was randomized. The experiment started after the eye calibration and the presentation of three practice items, two of which were followed by a comprehension question. Participants were instructed to read the stimulus texts carefully for meaning at their normal reading pace. The L1 speakers finished the experiment in approximately 45 and the L2 speakers in approximately 70 min. After completing the experiment each participant either received course credit or a small monetary compensation as a reward for their contribution.

The analysis regions of primary interest included the critical region containing the pronoun and the complementizer preceding it (*dass er* 'that he'), and the postcritical region consisting of the two words following the pronoun. We extended the pronoun region backwards by one word so as to be able to capture potential parafoveal viewings of the pronoun, which might be skipped during initial reading (e.g. Sturt, 2003). The following eye-movement measures were analysed: first-pass reading times (the summed duration of all fixations on a region of interest before exiting it to the left or right), right-bound reading times (the summed duration of all fixations on a region of interest before exiting it to the right), rereading times (the summed duration of all fixations on a region of interest after it has been exited to the left or to the right), and total reading times (the summed duration of all fixations on a region of interest), We also analysed the probabilities of first-pass regressions from, and of rereading, the two regions of interest.

The data were analysed in R version 3.6.0 (R Core Team, 2019) using mixed modelling with the lmerTest package version 3.1.2 (Kuznetsova et al., 2017). Linear-mixed effects models were fitted for the continuous measures and mixed-effects logistic regressions for the binomial dependent variables. For each region and measure, the effects of interest were inserted into one model. P-values were calculated from the model output rather than from model comparison. Given that the distribution of fixation durations is often right-skewed, we used the Box-Cox procedure (Box & Cox, 1964) to determine the appropriate data transformation for each of the two regions of interest. In consequence, a non-linear transformation (log) was applied to each reading-time continuous measure to satisfy the assumptions of normality. Statistical analyses were performed on the log-transformed data.

We first carried out a between-group analysis and subsequently, as several interactions with the factor Group reached significance, analysed the L1 group and the L2 group separately. The fixed parts of the models for the between-group analysis contained the sum-coded two-level factors QP (match, mismatch), DP (match, mismatch), and Group (L1, L2), the centred factor Trial, and their interactions. The random parts of the models contained, in their simplest version, by-subject and by-item random intercepts (Formula: log (value) ~ QP.sum * DP.sum * group.sum* c.(Trial) + (1|Subj) + (1|Item)). The within-group models contained the sum-coded two-level factors QP (match, mismatch) and DP (match, mismatch), centred Trial, and their interactions in the fixed parts and by-subject and by-item random intercepts in the simplest version of the random parts (Formula: log (value) ~ QP.sum * DP.sum * c.(Trial) + (1|Subj) + (1|Item)). To find out whether a more complex random structure provided a better fit to our data we conducted a series of models whose random parts contained different levels of complexity. The full random structures contained by-subject and by-item random slopes for QP, DP, and their interaction (+ (1 + QP.sum * DP.sum|Subj) + (1 + QP.sum * DP.sum|Item)). Likelihood ratio tests were applied to determine the adequate level of complexity. The models were refitted with full maximum likelihood estimation. We selected the simplest model unless a more complex one proved to be a significantly better fit at an alpha level of 0.05 (Baayen et al., 2008). In the vast majority of cases, this procedure led to the simplest model being selected (see the formulae above). On the rare occasion that convergence for the simplest structure could not be achieved, we removed first Trial, then Trial and the by-Item intercept, and lastly Trial and the by-Subject intercept. Trial refers to the order of item presentation. It was originally included to account for changes in the effects of the predictors over the course of the experiment due to, for example, tiredness (slow-down) or learning (speed-up). As a numeric variable that does not have a meaningful zero value, Trial was mean-centred. Since no interaction with this variable reached statistical significance, Trial will not be discussed any further.

#### Predictions

Participants' attempts to link the pronoun to a DP or QP antecedent should be reflected in corresponding gender-mismatch effects. If dependency formation is attempted during the initial reading of the pronoun region, we expect to find gender effects in early eye-movement measures, including first-pass and right-bound reading time. If, on the other hand, dependency formation with the DP or QP antecedent is only attempted at later processing stages, we expect to find gender effects restricted to later (rereading time and/or rereading probability) or composite measures (total viewing time), or to the postcritical region. If the QP antecedent is considered later than the DP antecedent or if it is not considered at all, then QP gender effects should be delayed or absent. This outcome would be expected if telescoping readings are only computed during later comprehension stages, or if pronouns that enter into telescoping dependencies are of a special type that is insensitive to gender congruence, as has been suggested by Moulton and Han (2018).

For both L1 and L2 processing, an antecedent search strategy that is based on surface syntax or which is discourse-based would lead comprehenders to focus on the DP antecedent in matrix subject position. If L2 speakers have more difficulty than L1 speakers computing non-isomorphic syntax-semantic mappings in real time, then QP gender effects should be delayed (relative to the L1 group) or altogether absent in this group.

### Results

Both groups' comprehension accuracy was high (L1: 96%, range: 80–100%; L2: 93%, range: 73–100%), confirming that participants read the stimulus items actively for meaning. During reading the L1 group skipped the critical region 14.6% and the postcritical one 4.5% of the time. Skipping rates for the L2 group were 6.5% for the critical and 2.8% for the postcritical region. One experimental item and one condition of another item were excluded because they contained an error. Individual fixations shorter than 40 ms or longer than 1000 ms were removed, comprising 0.50% of the L1 data and 0.45% of the L2 data. Individual trials excluded for track loss comprised 0.21% of the L1 data and 0.27% of the L2 data.

Table 3 provides an overview of the two participant groups' reading times across the four experimental conditions, and Table 4 shows the probabilities of first-pass regressions and of rereading the two interest regions. Table 5 shows our initial between-groups analysis that revealed main effects of, and interactions with the factor group, in several eye-movement measures and across both interest regions. Positive estimates for QP and DP indicate longer reading times/higher probabilities in the gender mismatch as compared to the gender match condition. Main effects of group were found in all continuous reading time measures and reflected the fact that the L2 group generally read more slowly than the L1 group. At the pronoun region, QP by Group interactions were found in first-pass and right-bound reading times. Marginally significant three-way interactions between QP, DP and Group were seen in total reading times at both interest regions. There was a further QP by Group interaction in participants' first-pass regressions at the postcritical region, and DP by Group interactions were found for rereading probability at both interest regions. As these results indicate group differences in participants' reading-time patterns across our experimental conditions, we went on to analyse the data from the two groups separately.

**Table 3:** Native (L1) and non-native (L2) speakers’ mean reading times in the pronoun and postcritical regions in milliseconds (SDs and 95% CIs in parentheses), Experiment 2

		Pronoun Region				Postcritical Region			
		1^st^ Pass Time	Right-bound Time	Rereading Time	Total Time	1^st^ Pass Time	Right-bound Time	Rereading Time	Total Time
L1	DP Match, QP Match (= 12a)	321 (192; 299–344)	327 (194; 305–350)	313 (202; 274–352)	397 (262; 368–426)	393 (233; 368–419)	441 (257; 413–470)	407 (302; 355–459)	549 (358; 510–588)
	DP Match, QP Mismatch (= 12b)	319 (155; 301–337)	333 (168; 313–352)	351 (230; 307–395)	394 (236; 369–419)	370 (205; 348–393)	415 (230; 391–440)	387 (278; 341–433)	525 (311; 492–558)
	DP Mismatch, QP Match (= 12c)	312 (162; 294–331)	328 (200; 305–350)	360 (291; 312–407)	442 (308; 409–475)	369 (209; 346–391)	426 (239; 400–452)	452 (347; 400–504)	585 (363; 546–623)
	DP Mismatch, QP Mismatch (= 12d)	341 (218; 316–367)	357 (235; 330–385)	453 (343; 403–504)	562 (419; 516–608)	385 (241; 358–411)	482 (296; 449–514)	489 (428; 430–549)	686 (457; 636–736)
L2	DP Match, QP Match (= 12a)	371 (193; 347–396)	389 (200; 363–414)	339 (224; 296–381)	500 (278; 466–535)	535 (292; 499–571)	605 (315; 566–644)	560 (365; 493–628)	781 (453; 725–837)
	DP Match, QP Mismatch (= 12b)	359 (197; 334–383)	373 (206; 347–398)	432 (337; 369–496)	525 (347; 483–567)	515 (258; 484–546)	556 (257; 525–587)	600 (418; 528–673)	797 (490; 738–856)
	DP Mismatch, QP Match (= 12c)	372 (206; 346–397)	385 (207; 360–411)	475 (365; 409–541)	571 (400; 523–619)	517 (301; 480–553)	587 (315; 549–625)	720 (569; 625–815)	880 (601; 808–952)
	DP Mismatch, QP Mismatch (= 12d)	359 (195; 334–383)	375 (205; 350–401)	541 (408; 468–615)	606 (438; 552–660)	513 (265; 480–546)	603 (332; 562–644)	717 (628; 610–823)	891 (622; 815–968)

**Table 4:** Native (L1) and non-native (L2) speakers’ proportions of first-pass regressions and of rereading the pronoun and postcritical regions (SEs in parentheses), Experiment 2

		Pronoun Region		Postcritical Region	
		First-pass Regression Probability	Rereading Probability	First-pass Regression Probability	Rereading Probability
L1	DP Match, QP Match (= 12a)	.03 (.01)	.30 (.02)	.13 (.02)	.38 (.03)
	DP Match, QP Mismatch (= 12b)	.04 (.01)	.31 (.02)	.16 (.02)	.42 (.03)
	DP Mismatch, QP Match (= 12c)	.04 (.01)	.42 (.03)	.17 (.02)	.50 (.03)
	DP Mismatch, QP Mismatch (= 12d)	.05 (.01)	.54 (.03)	.27 (.02)	.62 (.03)
L2	DP Match, QP Match (= 12a)	.04 (.01)	.41 (.03)	.13 (.02)	.43 (.03)
	DP Match, QP Mismatch (= 12b)	.04 (.01)	.41 (.03)	.12 (.02)	.48 (.03)
	DP Mismatch, QP Match (= 12c)	.04 (.01)	.45 (.03)	.17 (.02)	.52 (.03)
	DP Mismatch, QP Mismatch (= 12d)	.04 (.01)	.47 (.03)	.15 (.02)	.53 (.03)

**Table 5:** Summary of the statistical between-group analyses of the eye-movement data for the two interest regions, Experiment 2

	Critical region				Postcritical region			
	Estimate	SE	*t* (*z*)	*p*	Estimate	SE	*t* (*z*)	*p*
*First-pass reading times*								
Intercept	**5.701**	**0.029**	**194.6**	** < 0.001**	**5.945**	**0.036**	**165.5**	** < 0.001**
QP	0.003	0.009	0.402	0.688	0.001	0.011	0.050	0.960
DP	0.007	0.009	0.761	0.447	−0.013	0.023	−0.552	0.586
Group	**−0.067**	**0.027**	**−2.499**	**0.014**	**−0.164**	**0.025**	**−6.439**	** < 0.001**
QP*DP	0.005	0.009	0.525	0.600	0.014	0.010	1.455	0.146
QP*Group	**0.019**	**0.009**	**2.173**	**0.030**	−0.007	0.010	−0.674	0.500
DP*Group	0.003	0.009	0.360	0.720	0.003	0.011	0.279	0.781
QP*DP*Group	0.007	0.009	0.770	0.441	0.003	0.010	0.340	0.734
*Right-bound reading times*								
Intercept	**5.739**	**0.030**	**191.4**	** < 0.001**	**6.093**	**0.043**	**141.5**	** < 0.001**
QP	0.006	0.009	0.691	0.490	0.007	0.010	0.670	0.505
DP	0.008	0.009	0.915	0.360	0.013	0.028	0.473	0.641
Group	**−0.073**	**0.028**	**−2.651**	**0.009**	**−0.158**	**0.026**	**−5.987**	** < 0.001**
QP*DP	0.005	0.009	0.626	0.532	**0.029**	**0.009**	**3.315**	**0.001**
QP*Group	**0.024**	**0.009**	**2.728**	**0.006**	0.009	0.010	0.891	0.375
DP*Group	0.005	0.009	0.538	0.591	0.008	0.010	0.805	0.423
QP*DP*Group	0.003	0.009	0.313	0.755	0.008	0.009	0.963	0.337
*Rereading times*								
Intercept	**5.742**	**0.039**	**147.1**	** < 0.001**	**5.975**	**0.038**	**158.7**	** < 0.001**
QP	**0.072**	**0.022**	**3.339**	**0.002**	−0.004	0.024	−0.190	0.850
DP	**0.104**	**0.026**	**3.960**	**0.001**	**0.078**	**0.030**	**2.621**	**0.015**
Group	**−0.075**	**0.033**	**−2.256**	**0.026**	**−0.180**	**0.029**	**−6.131**	** < 0.001**
QP*DP	0.002	0.019	0.093	0.926	−0.006	0.020	−0.275	0.784
QP*Group	−0.013	0.021	−0.621	0.536	0.009	0.021	0.420	0.675
DP*Group	−0.026	0.021	−1.218	0.227	−0.012	0.022	−0.565	0.573
QP*DP*Group	0.027	0.019	1.439	0.150	0.013	0.020	0.654	0.513
*Total reading times*								
Intercept	**6.008**	**0.036**	**164.6**	** < 0.001**	**6.371**	**0.043**	**147.1**	** < 0.001**
QP	**0.035**	**0.012**	**2.947**	**0.005**	0.016	0.011	1.426	0.159
DP	**0.073**	**0.016**	**4.487**	** < 0.001**	**0.058**	**0.027**	**2.159**	**0.041**
Group	**−0.106**	**0.033**	**−3.226**	**0.002**	**−0.174**	**0.027**	**−6.313**	** < 0.001**
QP*DP	0.025	0.012	2.052	0.052	0.019	0.012	1.603	0.112
QP*Group	0.018	0.011	1.603	0.112	0.010	0.011	0.914	0.363
DP*Group	0.021	0.011	1.934	0.054	0.017	0.012	1.474	0.144
QP*DP*Group	0.018	0.011	1.706	0.089	0.020	0.012	1.721	0.088
*First-pass regression probability*								
Intercept	**−3.136**	**0.103**	**−30.53**	** < 0.001**	**−1.931**	**0.110**	**−17.49**	** < 0.001**
QP	0.086	0.103	0.832	0.405	0.086	0.059	1.446	0.148
DP	0.069	0.103	0.672	0.502	**0.213**	**0.059**	**3.576**	** < 0.001**
Group	−0.036	0.103	−0.348	0.728	0.185	0.106	1.746	0.081
QP*DP	0.003	0.103	0.030	0.976	0.049	0.059	0.832	0.405
QP*Group	0.118	0.103	1.150	0.250	**0.155**	**0.059**	**2.608**	**0.009**
DP*Group	0.060	0.103	0.584	0.559	0.051	0.059	0.859	0.390
QP*DP*Group	−0.010	0.103	−0.095	0.925	0.063	0.059	1.056	0.291
*Rereading probability*								
Intercept	**−0.420**	**0.107**	**−3.942**	** < 0.001**	−0.078	0.116	−0.674	0.500
QP	0.087	0.046	1.902	0.057	**0.138**	**0.045**	**3.050**	**0.002**
DP	**0.273**	**0.046**	**5.961**	** < 0.001**	**0.276**	**0.045**	**6.077**	** < 0.001**
Group	−0.094	0.098	−0.954	0.340	−0.026	0.100	−0.261	0.794
QP*DP	0.079	0.046	1.733	0.083	0.023	0.045	0.504	0.614
QP*Group	0.058	0.045	1.283	0.199	0.056	0.045	1.242	0.214
DP*Group	**0.157**	**0.046**	**3.435**	**0.001**	**0.106**	**0.045**	**2.346**	**0.019**
QP*DP*Group	0.055	0.045	1.201	0.230	0.072	0.045	1.586	0.113

*p* < .05 in bold

Table 6 shows the model outputs for the L1 group. At the critical region, significant main effects of QP gender were found in all reading-time measures reported. They reflect the fact that reading times were shorter if the QP's gender matched the gender of the pronoun compared to when it did not.

**Table 6:** Summary of the statistical analyses of the L1 group's eye-movement data for the two interest regions, Experiment 2

	Critical region				Postcritical region			
	Estimate	SE	*t* (*z*)	*p*	Estimate	SE	*t* (*z*)	*p*
*First-pass reading times*								
Intercept	**5.633**	**0.039**	**144.1**	** < 0.001**	**5.781**	**0.043**	**134.6**	** < 0.001**
QP	**0.023**	**0.011**	**2.079**	**0.038**	−0.007	0.015	−0.454	0.652
DP	0.010	0.011	0.924	0.356	−0.010	0.022	−0.439	0.664
QP*DP	0.012	0.011	1.107	0.269	0.018	0.013	1.382	0.167
*Right-bound reading times*								
Intercept	**5.665**	**0.041**	**137.0**	** < 0.001**	**5.934**	**0.051**	**116.0**	** < 0.001**
QP	**0.030**	**0.011**	**2.740**	**0.006**	0.013	0.014	0.911	0.067
DP	0.013	0.011	1.175	0.240	0.019	0.028	0.674	0.506
QP*DP	0.009	0.011	0.777	0.437	**0.037**	**0.012**	**3.094**	**0.002**
*Rereading times*								
Intercept	**5.675**	**0.046**	**123.7**	** < 0.001**	**5.814**	**0.036**	**162.5**	** < 0.001**
QP	**0.063**	**0.027**	**2.351**	**0.019**	0.006	0.027	0.224	0.823
DP	**0.073**	**0.027**	**2.746**	**0.006**	**0.064**	**0.027**	**2.407**	**0.016**
QP*DP	0.031	0.027	1.144	0.253	0.011	0.027	0.424	0.672
*Total reading times*								
Intercept	**5.903**	**0.047**	**126.5**	** < 0.001**	**6.199**	**0.048**	**130.3**	** < 0.001**
QP	**0.055**	**0.014**	**4.044**	** < 0.001**	0.025	0.015	1.701	0.096
DP	**0.095**	**0.014**	**6.995**	** < 0.001**	**0.075**	**0.028**	**2.702**	**0.013**
QP*DP	**0.044**	**0.014**	**3.252**	**0.001**	**0.039**	**0.013**	**2.957**	**0.003**
*First-pass regression probability*								
Intercept	**−3.494**	**0.234**	**−14.90**	** < 0.001**	**−1.780**	**0.153**	**−11.61**	** < 0.001**
QP	0.210	0.142	1.486	0.137	**0.237**	**0.075**	**3.153**	**0.001**
DP	0.140	0.142	0.990	0.322	**0.267**	**0.076**	**3.533**	** < 0.001**
QP*DP	−0.006	0.141	−0.045	0.964	0.107	0.075	1.428	0.153
*Rereading probability*								
Intercept	**−0.520**	**0.136**	**−3.817**	** < 0.001**	−0.107	0.152	−0.705	0.481
QP	**0.155**	**0.063**	**2.470**	**0.014**	**0.195**	**0.061**	**3.209**	**0.001**
DP	**0.430**	**0.062**	**6.972**	** < 0.001**	**0.340**	**0.061**	**6.366**	** < 0.001**
QP*DP	**0.122**	**0.062**	**1.985**	**0.047**	0.091	0.061	1.487	0.137

*p* < .05 in bold

Main effects of DP gender, reflecting shorter reading times for DP-match than for DP-mismatch conditions, were restricted to later or composite eye-movement measures including rereading and total reading times, and were also found for rereading probability. QP by DP interactions were found for rereading probability and total reading times. Pairwise comparisons showed that Gender-mismatching QPs led to a significantly higher probability of participants rereading the pronoun region when no gender-matching DP was available (Est. = 0.271, *z* = 3.223, *p* = 0.001) but not when a gender-matching DP was available (Est. = 0.017, *z* = 0.191, *p* = 0.848). Similarly, mismatching QPs elicited elevated total reading times in the DP-mismatch conditions (Est. = 0.099, *z* = 4.745, *p* < 0.001) but not in the DP-match conditions (Est. = 0.011, *t* = 0.616, *p* = 0.538).

At the postcritical region, the L1 group showed main effects of QP gender in both first-pass regressions and rereading probability, and main effects of DP gender in first-pass regressions, rereading probability, and in rereading and total viewing times. The two factors interacted in right-bound and total reading times. The pattern was the same as we observed at the critical region: Pairwise comparisons revealed that Gender-mismatching QPs led to significantly higher reading times when no matching DP antecedent was available (right-bound time: Est. = 0.050, *t* = 2.892, *p* = 0.004; total reading time: Est. = 0.065, *t* = 3.361, *p* = 0.001) but not when a matching DP antecedent was available (right-bound time: Est. = −0.026, *t* = −1.677, *p* = 0.094; total reading time: Est. = -0.014, *t* = -0.744, *p* = 0.457).

The model output for the L2 group's data is shown in Table 7. For this group, no significant effects or interactions were found in early reading-time measures (i.e., in first-pass or right-bound reading times). At the pronoun region, significant or marginal effects of DP gender were seen in rereading and total reading times, as well as in rereading probability. A main effect of QP gender was found in rereading times only, and there were no interactions. At the postcritical region, only DP gender showed any significant effects, which were again restricted to later eye-movement measures (i.e. rereading times and rereading probability).

**Table 7:** Summary of the statistical analyses of the L2 group's eye-movement data for the two interest regions, Experiment 2

	Critical region				Postcritical region			
	Estimate	SE	*t* (*z*)	*p*	Estimate	SE	*t* (*z*)	*p*
First-pass reading times								
Intercept	**5.768**	**0.040**	**143.7**	** < 0.001**	**6.111**	**0.048**	**126.6**	** < 0.001**
QP	−0.016	0.014	−1.182	0.237	0.009	0.016	0.581	0.563
DP	0.003	0.014	0.205	0.838	−0.015	0.030	−0.487	0.631
QP*DP	−0.002	0.014	−0.131	0.896	0.013	0.015	0.923	0.356
Right-bound reading times								
Intercept	**5.813**	**0.039**	**149.4**	** < 0.001**	**6.251**	**0.051**	**123.7**	** < 0.001**
QP	−0.018	0.013	−1.350	0.177	0.001	0.016	0.067	0.947
DP	0.003	0.013	0.210	0.833	0.007	0.031	0.235	0.816
QP*DP	0.003	0.013	0.232	0.817	0.022	0.014	1.556	0.127
Rereading times								
Intercept	**5.821**	**0.058**	**99.78**	** < 0.001**	**6.138**	**0.059**	**103.3**	** < 0.001**
QP	**0.087**	**0.028**	**3.100**	**0.002**	−0.002	0.031	−0.049	0.961
DP	**0.123**	**0.028**	**4.399**	** < 0.001**	**0.106**	**0.031**	**3.402**	**0.001**
QP*DP	−0.022	0.028	−0.788	0.431	−0.023	0.031	−0.749	0.454
Total reading times								
Intercept	**6.114**	**0.052**	**118.1**	** < 0.001**	**6.544**	**0.057**	**115.4**	** < 0.001**
QP	0.015	0.016	0.931	0.352	0.007	0.018	0.407	0.686
DP	**0.051**	**0.016**	**3.139**	**0.002**	0.042	0.030	1.400	0.174
QP*DP	0.008	0.016	0.514	0.608	−0.001	0.015	−0.074	0.941
First-pass regression probability								
Intercept	**−3.108**	**0.152**	**−20.46**	** < 0.001**	**−1.835**	**0.089**	**−20.54**	** < 0.001**
QP	−0.032	0.151	−0.210	0.834	−0.062	0.089	−0.692	0.489
DP	0.008	0.151	0.054	0.957	0.150	0.089	1.684	0.092
QP*DP	0.015	0.151	0.102	0.919	−0.009	0.089	−0.104	0.917
Rereading probability								
Intercept	**−0.329**	**0.163**	**−2.018**	**0.044**	−0.054	0.160	−0.336	0.737
QP	0.030	0.068	0.438	0.661	0.079	0.068	1.172	0.241
DP	0.117	0.068	1.720	0.085	**0.171**	**0.068**	**2.528**	**0.011**
QP*DP	0.025	0.068	0.366	0.714	−0.055	0.068	−0.814	0.416

*p* < .05 in bold

In summary, our two participant groups showed very different reading-time patterns across the four experimental conditions. The L1 group showed main effects of QP gender in early reading-time measures at the pronoun region, as well as in later eye-movement measures and across both regions of interest. Effects of DP gender only appeared in later measures in the critical region and in the postcritical region. The interactions observed in some later measures and at the postcritical region showed QP-mismatch effects being restricted to the DP-mismatch conditions. The L2 group, in contrast, showed no early effects of either antecedent's gender but showed robust effects of DP gender in later eye-movement measures, and no effects of the QP's gender except during their rereading of the pronoun region.

### Discussion

In Experiment 2 we asked whether L1 and/or L2 speakers of German would try to link a pronoun to a non c-commanding object QP during processing. The early main effects of QP gender, in the absence of any effects of DP gender, that were seen in the L1 group indicate that the native German speakers primarily considered the QP antecedent during their initial reading of the pronoun. QP effects persisted across several eye-movement measures and both interest regions, indicating that telescoping dependencies were attempted from early on during processing. Effects of the DP antecedent's gender became visible only with some delay. The L1 speakers' eye-movement record shows that they gradually homed in on the DP antecedent over time, but with QP antecedents still being considered if the DP antecedent mismatched the pronoun in gender. A reviewer points out that the interaction pattern we observed in total reading times and rereading probability in the L1 data resembles a faciliatory interference pattern (Jäger et al., 2017). Note, however, that the present study was not designed to test memory interference as our stimulus sentences contained two permissible antecedents and no inappropriate distractor. Hence neither consideration of the DP nor of the QP antecedent can serve as a reliable diagnostic for interference here.

As noted above, our L1 group's results contrast with the absence of QP gender effects in previous processing experiments (Cunnings et al., 2015; Kush et al., 2015; Moulton & Han, 2018). To our knowledge, the current study is the first to observe gender-mismatch effects for non c-commanding QP antecedents. One possible reason for this is that previous studies used stimulus materials with QPs in subject position, whereas we used QPs in object position. Syntactic approaches to telescoping out of RCs which assume that the QP must undergo QR in order to take scope over the pronoun would account for this apparent subject/object asymmetry in terms of a grammatical constraint which prohibits or penalises the extraction of subjects. This account must remain speculative, though, as we did not compare sentences with subject and object QPs directly (as did Radó et al., 2019).

Our L2 group, in contrast, did not show any evidence of considering the QP antecedent during processing, except fleetingly during their rereading of the pronoun region. Instead, the L2 speakers showed robust effects of DP gender in later eye-movement measures across both interest regions, suggesting that they tried to resolve the pronoun towards the DP antecedent. The L2 group's reading-time pattern is in line with previous findings indicating that L2 speakers prefer to link pronominal elements to discourse-prominent antecedents during processing and avoid quantified antecedents even if they are potential binders.

## General Discussion

Taken together, our results show that both L1 and L2 comprehenders can obtain bound readings for pronouns in the absence of a c-command relation, but only L1 comprehenders attempt to link pronouns to QPs inside relative clauses during incremental parsing. The results from Experiment 1 are consistent with previous findings showing that telescoping readings are readily available to L1 comprehenders (Carminati et al., 2002; Moulton & Han, 2018; Radó et al., 2019) and extend this finding to L2 comprehension. The observed subject/object asymmetries in participants' responses furthermore suggest that pronouns can be linked more easily to embedded objects than to embedded subjects. This would be expected from the perspective of syntactic approaches to telescoping out of RCs, but since we observed a parallel asymmetry for DP antecedents, this finding should be interpreted with some caution.

In Experiment 2, the distribution of QP effects in our L1 speakers' eye-movement record indicates that the L1 group considered a bound variable reading of the pronoun from early on during processing and before considering the alternative DP antecedent. This is a novel finding as previous reading-time studies did not observe any gender effects for non c-commanding QPs (Cunnings et al., 2015; Kush et al., 2015; Moulton & Han, 2018). Given the results from Experiment 1, it is conceivable that object QPs can scope out of RCs more readily than subject QPs, which would account for the lack of QP effects in previous processing studies using subject QPs. Our eye-movement results furthermore show that there is no reason to think that pronouns entering into a telescoping dependency are of a different type from other bound pronouns (cf. Moulton & Han, 2018), at least not as far as their sensitivity to gender congruence is concerned.

Although the present study was not designed to test memory interference accounts of pronoun resolution, it may be worth considering whether the QP by DP interactions seen in the L1 speakers' eye-movement data may reflect interference. We do not think that interference provides a plausible explanation for this finding, however, for the following reasons: First, recall that previous studies on telescoping found evidence for telescoping readings in offline measures but no QP effects in online measures, which is the opposite of what we might expect if non c-commanding QPs trigger interference when a pronoun is encountered. Secondly, the fact that object QPs (in the current study) but not subject QPs (Cunnings et al., 2015; Kush et al., 2015; Moulton & Han, 2018) are considered during processing has no straightforward explanation in terms of memory interference but is predicted by syntactic accounts of telescoping. That said, cue-based retrieval models of pronoun resolution might be able to account for the subject/object asymmetry depending on how retrieval cues are weighted. However, assuming that for subject pronouns, antecedent subjecthood is a strong cue, cue-based models would seem to predict the opposite of what has been found across different studies. Thirdly, if the QP by DP interaction in the L1 data reflected interference, it is unclear why our L2 speakers should not have been affected by an intervening QP in the same way, or indeed more strongly, given that L2 speakers have been claimed to be more prone to interference than L1 speakers (Cunnings, 2017). A statistical interaction pattern characteristic of interference is altogether absent from the L2 group's eye-movement data, however.

Unlike our L1 group, the L2 participants did not show any evidence of trying to link the pronoun to a non c-commanding QP during their initial reading of the critical and postcritical regions. Their eye-movement record indicates that they tried to resolve the pronoun towards the matrix subject instead. This finding is consistent with previous eye-movement results suggesting that L2 comprehenders are drawn towards discourse-prominent antecedents during anaphor resolution even if a less prominent alternative antecedent (Patterson et al., 2014) or a local binder (Felser & Cunnings, 2012) is available. In the current study the L2 group's focusing on the DP antecedent indicates an antecedent search strategy that is either based on surface syntax, according to which the QP is not a possible binder, or on discourse-level cues such as the DP antecedent's topicality. A discourse-based antecedent search strategy would also disfavour the QP antecedent due to its non-referentiality or conceptual number mismatch with the singular pronoun.

Considering that our L2 speakers were able to obtain telescoping readings if given more time, we suggest instead that the L1/L2 differences we observed in Experiment 2 reflect L2 comprehenders' difficulty manipulating previously built structural representations (e.g. Felser, 2016, 2019). On the assumption that computing telescoping readings requires the QP's scope to be extended, our L2 comprehenders might not have been able to perform this kind of non-isomorphic syntax-semantics mapping in real time (compare also Boxell et al., 2017). This could be because syntactic representations are not built quickly enough, lack sufficient detail, or are less stable in L2 than in L1 processing. For our stimulus sentences, an antecedent search which relies on surface-syntactic and/or discourse-level cues provides an alternative way of identifying an antecedent. In highly proficient L2 speakers as were examined in the present study, difficulty establishing complex form-to-meaning mappings might only be detectable by using time-course sensitive experimental methods.

L2 speakers showing native-like behaviour in offline tasks (Experiment 1) but non-nativelike processing patterns (Experiment 2) is not an unusual finding in L2 processing research (compare e.g. Felser & Cunnings, 2012). Experimental tasks that tap into comprehenders' initial preferences may yield different results from tasks which allow for conscious reasoning or deliberation. Being given time to evaluate our stimulus sentences and the requirement to provide an explicit response clearly led our L2 comprehenders to admit telescoping antecedents. Note that telescoping interpretations have been argued to be derivable without covert syntactic movement (e.g. Sternefeld, 2019). That is to say, the same outcome—here, telescoping—may potentially be achieved via different interpretive routines, with the possibility that a given routine is more likely to be used by one population than by another.

Future research might want to investigate potential subject/object asymmetries in telescoping more systematically to test the predictions derived from different formal approaches to telescoping. Examining other L1/L2 combinations, and the processing of other kinds of syntax-semantics mismatches, would be useful in order to further explore the potential limits of advanced L2 comprehenders' processing abilities.

## Conclusion

In contrast to what has been reported in previous reading-time studies on telescoping, our eye-movement results provide evidence of native speakers being able to derive these readings not only offline but also during real-time processing. A possible reason for this discrepancy is that object QPs, which we used in the current study, undergo scope-changing operations more easily than the subject QPs used in previous studies, but more research is required to test this hypothesis. Although the L2 speakers we examined were able to obtain telescoping readings offline, they did not show any evidence of deriving these during online processing, focusing on a discourse-prominent, non-quantified antecedent instead. We interpret this finding as reflecting difficulty manipulating structural representations during non-native language processing and L2 comprehenders following a surface structure or discourse-based antecedent search strategy instead. The observed L1/L2 differences provide evidence that arriving at the same ultimate interpretations does not necessarily imply the use of the same online processing routines.

## Data Availability

A full list of our experimental materials, statistical model outputs for our between-groups analyses, and an overview of the formulae that were used for modelling the results of Experiment 2 are available at the OSF website at https://osf.io/b3duv/.

## Footnotes

1 A syntactic constituent c-commands its sister constituents and all nodes in a phrase marker that its sister constituents dominate (Reinhart, 1983).

2 There is no need for non-quantificational antecedents to undergo QR as these can be linked to a pronoun via coreference assignment in the absence of c-command (e.g. Grodzinsky & Reinhart, 1993).

3 The ECP was proposed within government and binding theory (Chomsky, 1981) to account for subject-object asymmetries in extraction. In more recent versions of generative grammar, ECP effects are accounted for by other formal constraints (compare e.g. Rizzi, 2016).

4 As Russian L1 speakers have been shown to be able to compute scope-shifted readings (Antonyuk, 2019), their ability to obtain telescoping readings in L2 German should not be affected by negative L1 influence.
